# Middle-range theory for men’s health promotion: contributions to sustainable development

**DOI:** 10.1590/0034-7167-2025-0216

**Published:** 2026-02-23

**Authors:** Isabele Torquato Mozer Rosa, Aurea Christina de Paula Corrêa, Nuno Damácio de Carvalho Félix, Jeane Cristina Anschau Xavier Oliveira

**Affiliations:** I Universidade Federal de Mato Grosso. Cuiabá, Mato Grosso, Brazil.; II Universidade Federal do Recôncavo Baiano. Santo Antônio de Jesus, Bahia, Brazil.

**Keywords:** Nursing Theory, Health Promotion, Men’s Health, Masculinity, Concept Formation

## Abstract

**Objectives:**

to develop a middle-range nursing theory for men’s health promotion aligned with the gender equality Sustainable Development Goal.

**Methods:**

an inductive-deductive theoretical study, analyzing the Health Promotion Model, conceptual analysis, purpose, propositions, assumptions, and modeling. The empirical basis was drawn from the national survey on the Brazilian National Policy for Comprehensive Men’s Health Care from a gender equality perspective.

**Results:**

known as the Lighthouse Theory, the theory is composed of 17 concepts, ten propositions, and four assumptions, represented in a model, with the purpose of describing men’s health promotion, culminating in an action plan for healthy behaviors, aiming for equality in health care.

**Final Considerations:**

the Lighthouse Theory offers an innovative theoretical framework for nursing and health, strengthening men’s health promotion practices by describing specific behaviors.

## INTRODUCTION

The issue of men’s health (MH) and masculinities was presented as a commitment of the Pan American Health Organization (PAHO) in the 2014-2019 Strategic Plan^(1)^, and continues to be a focal point in actions towards universal health and gender equality. In this context, gender equality must also involve men and their healthcare, as they are considered part of the solution to such inequalities. In a recent publication, this issue was included in the “health throughout the life course” component of the aforementioned plan, for the years 2020 and 2025, also aligned with the 2030 Agenda for Sustainable Development^(2)^. Thus, the development of theoretical knowledge aligns with the fifth Sustainable Development Goal (SDG) (“Gender equality”), with potential benefits in presenting pathways for promoting MH.

Nursing is considered an important professional body for achieving the SDGs. The concern for improving nursing training quality, focusing on the needs of health systems, access, and universal health coverage, reinforces the importance of this discipline in achieving the SDGs^(3)^. The union of two investment areas of this health organization, the foundation for public health in the Americas, provides countless benefits for MH promotion.

In the national context, MH stood out as a public policy after establishing the Brazilian National Policy for Comprehensive Men’s Healthcare (in Portuguese, *Política Nacional de Atenção Integral à Saúde do Homem* - PNAISH) in 2009, and since then, it has remained the only one in the Americas^(4)^. The discussion of masculinities and their relationship with clinical and epidemiological issues offers a proposal, albeit tentative, yet innovative for promoting and restoring this population’s health. Fifteen years after its creation, the inclusion of men in services as a focus on health promotion and the provision of actions aimed at this population remain challenging tasks for healthcare professionals, requiring the structuring of disciplinary knowledge to describe the scope of practice, especially in nursing. Typically, nursing actions for men’s health are still limited to prostate cancer prevention and treatment in specific campaigns^(5)^.

Internationally, men are predominantly involved in sexually transmitted infection testing and prenatal care. Nurses still do not recognize men as subjects of their care due to a lack of knowledge about male reproduction^(6)^. Evidence suggests that nurses consider their understanding of the concept and definition of men’s health to be insufficient, stemming from deficiencies in training. Such situations affect their confidence in providing care to men in this healthcare setting, also reflecting on men’s perceptions of the role of nurses in healthcare services^(7)^.

There is a lack of studies in national and international literature addressing the development of theoretical knowledge for nursing practice focused on MH care, exposing gaps in knowledge about the relationship between health promotion (HP), MH, and nursing. Professional practice is consistent with the findings in the literature, highlighting the need for a theoretical framework to support nursing practices for men in the context of MH.

In this context, nursing theories are the foundations of fundamental nursing and can, but should, serve as a resource for demonstrating paths that guide professional nursing practice within the context of HP. Regarding the development of nursing theories for this population group, there is a gap in knowledge within the disciplinary field of nursing, especially regarding middle-range theories (MRTs), which are considered a means to address the challenge of developing a body of knowledge specific to the discipline that guides nursing practice in various contexts^(8)^. This is reflected in the present study object, which encompasses concepts that address the specificities and subjectivities related to MH promotion, potentially contributing to guiding practice, teaching, and management in nursing and healthcare services.

## OBJECTIVES

To develop a nursing MRT to promote MH aligned with the SDG of gender equality.

## METHODS

### Ethical aspects

Submission to the Research Ethics Committee was waived, as this was a theoretical development study, but the copyright of cited publications was respected, in accordance with Law 9610/1998, which regulates such practices.

### Theoretical framework

Nola Pender’s Health Promotion Model (HPM) proposes identifying factors that influence healthy behaviors to explore the biopsychosocial processes that motivate individuals to engage in health behaviors. The HPM is based on social cognitive theory, arguing that the HPM promotes lifestyles and behaviors that enable people to maximize their potential through individual, organizational, and community changes, with nurses acting as catalysts for these processes. It is composed of the interrelation of three dimensions: individual characteristics and experiences; feelings and knowledge about specific behavior; and desired health behavior^(9)^.

### Study design

This is a theoretical development study, descriptive and basic in nature^(10)^, using deductive-inductive reasoning, based on empirical data from the Brazilian National Men’s Health Survey for induction and based on the HPM for deduction, generating, through the synthesis process, a new theory. This theory is the result of a doctoral thesis from the *Universidade Federal do Mato Grosso*, with Brazilian men and their health behaviors as a scenario^(11)^.

### Methodological procedures

The operationalization of this study was carried out in four phases, as described by Meleis^(12)^: 1) choosing a theoretical model to explain the phenomenon of interest; 2) redefining the theory’s concepts; 3) theoretical synthesis; and 4) presenting the new theory. This entire process is mental and requires creativity, abstraction, and reflection from the theorist. Problem-solving thinking, based on the interpretation and synthesis of findings, provides intuitive leaps that will aid in the development of the theory and its components.

The theoretical model chosen to explain the phenomenon of interest was the HPM, as it understands that there are analogous dimensions between the phenomenon of HP and MH promotion. To this end, Jacqueline Fawcett’s nursing model analysis framework was used^(13)^. This type of analysis is a detailed, nonjudgmental examination of the theory, which includes determining the theory’s origins, its unique focus, and its content, such as concepts, statements, model, and assumptions. For this purpose, the literary work “Health Promotion in Nursing Practice”, by Nola Pender, Carolyn Murdaugh, and Mary Ann Parsons, was used.

From the apprehension of a theoretical structure for support, the second phase began, redefining the theory concepts, carried out through: a) analysis of the concept according to Walker and Avant^(14)^ for the central concept of the “MH promotion” theory; b) conceptual derivation of the other concepts of the HPM according to Walker and Avant^(14)^. To this end, empirical data from the most recent national survey conducted with Brazilian men were used^(15)^. Theorizing these data allowed the multifaceted population of Brazilian men to be given a voice, in contrast to the development of PNAISH, in which formulators were not part of the primary target audience—the male population. The theory’s elements and concepts derive from the interpretations of men’s statements, demonstrating their understanding of HP and its needs, and particularly valuing their concerns about healthcare.

The third phase, theoretical synthesis, was carried out using the theoretical synthesis strategy^(14)^ due to the empirical evidence base, whose objective was to integrate the previously redefined concepts into a coherent theoretical framework. Thus, the concepts determined in the previous stage served as anchors for establishing their relationships based on the affirmation of associations and/or causality. This process involved the formulation of relational propositions and the explanation of assumptions^(14)^.

Relational propositions were developed to describe the interactions between the central concepts of the Lighthouse Theory. To this end, the following stages were performed: a) identification of potential relationships based on the empirical data analyzed, identifying patterns and associations between the previously defined concepts; b) formulation of propositions based on the identified relationships using clear and precise language. Each proposition was structured to reflect the nature of the relationship (causal, correlational, associative) between the concepts involved; c) theoretical validation by verifying these relationships with existing literature to examine their consistency and theoretical plausibility.

As propositions were formulated, insights into assumptions were recorded, being structured based on Walker and Avant’s guidelines^(14)^. To this end, the implicit beliefs that guided data interpretation and proposition formulation were identified, and then written as statements outlining the premises accepted as true within the context of the theory. This ensured that the assumptions were consistent with the theory concepts and propositions, avoiding contradictions or inconsistencies. Finally, ten assumptions were established—statements accepted as true—that establish the basic conditions under which the theory operates.

The theory pictogram was created taking as reference the determining elements for male HP, and was developed with the help of an online visual communication design platform, with the aim of illustrating nursing practice in the context of promoting MH.

## RESULTS

### Theory contextualization

The MRT for promoting health, known as the Lighthouse Theory, received this name for two reasons. The first relates to the biological factors that make men unique, associated with masculinities. The expressions of being a man in the collective environment are the composition of the light that should illuminate health-promoting behaviors and necessarily reflect on others. Masculinities and biological factors are already considered important concepts in the literature on health. However, envisioning their association with other concepts, as proposed by the Lighthouse Theory, aims to clarify for the nursing discipline a new way of conducting health-promoting practices for men, especially at the individual level, thus becoming the second reason.

### Theory purposes

Lighthouse Theory describes MH promotion, the relationship between behaviors that are harmful to health and masculinities, perceived courage, and its influence on perceived benefits and barriers, culminating in an action plan involving men’s and nursing professionals’ participation in healthy behavior promotion, with a view to equality in healthcare.

### Theory propositions and modeling

MH promotion is an ongoing process that can be mediated by healthcare professionals based on the recognition of men’s motivating elements, which are related to the environment’s expressions of masculinity. This is the central concept, whose definition is derived from the HPM, aligned with the nursing metaparadigm concepts and clarified by conceptual analysis. These concepts are presented as associated concepts, as described in Chart 1.

**Chart 1:** Propositions of middle-range theory for promoting men’s health, Cuiabá, Mato Grosso, Brazil, 2024

Concepts	Propositions
**Central concept**	
**Men’s health promotion**	Health information-seeking behaviors through healthcare services and healthcare professionals, other people of reference, and online media, aiming to improve well-being, prevent injuries and diseases, and practice/maintain self-care practices. These practices include adequate nutrition, regular physical activity, hydration, hygiene and rest, environmental forecasts, practices linked to culture and religion, aesthetic and physical appearance care, low alcohol intake, no tobacco, safe sex practices, an up-to-date vaccination schedule, attentive attention to mental health issues, and leisure time.
**Metaparadigmatic concepts**	
**Person**	A human being who identifies as male. They are shaped by their lifelong experiences within the context of family, school, work, and study, religion, culture, and online interactions. Individual characteristics, life experiences, peer behavior, and positive reinforcement received from a partner influence the shaping of health behaviors.
**Environment**	Social, cultural, physical and virtual context in which the course of men’s lives unfolds, manipulated/worked by the individual to create a positive context of suggestions and measures that will facilitate behaviors to improve their health, especially when they resort to spaces that involve other men they admire.
**Nursing**	A profession that provides support for the transformation of men’s health behaviors, working with men, women, families, and the community in which they are inserted, provided they are willing. This work aims to create an environment with favorable conditions for the expression of male health and well-being, and a judgmental role is not recommended.
**Health**	At the individual level, it is a sense of well-being acquired through health-promoting behaviors, involving the perception of one’s usefulness in the environment in which they live. Health information, whether obtained online or through healthcare professionals, can enhance or diminish this sense of well-being.
**Associated concepts**	
**Illness**	Events, whether acute or chronic, throughout the life cycle can encourage health-promoting behaviors in men.
**Biological factors**	Age, Body Mass Index, agility, hormonal status, personal and family health history.
**Masculinities**	Behaviors that identify being a man in society and give significant importance to existing behavior and new behavior are influenced by factors such as educational level, socioeconomic status, race/ethnicity, and psychological factors.
**Behaviors that are harmful to health**	Attitudes that must be changed for health and well-being, composed of biological factors, which cannot be changed, and masculinities. They should be interpreted as foundations of male behaviors related to health promotion and that must/need to be changed. They are expressed graphically as a beacon, as their light resonates with other concepts, with courage perceived as the first and most important attribute to the male population.
**Perceived courage**	Judging one’s own abilities to cope with emotionally or morally difficult situations and change unhealthy behavior. This judgment is directly influenced by masculinities, and therefore, establishing which benefits, barriers, feelings, and influences are most important for men.
**Perceived benefits**	Mental representations of the positive or reinforcing consequences of a behavior, characterized by well-being (having and maintaining a healthy life and feeling good), self-care (having the physical and mental conditions to take care of oneself), being a provider (perception of financially providing for oneself and/or family), autonomy (competence to manage one’s own life and make decisions), sense of usefulness (perception of being able to solve something for oneself or for someone else) and follow-up of descendants (actively participating in the growth and development of children and grandchildren).
**Perceived barriers**	Obstacles perceived by men in adopting health-promoting behaviors, characterized by financial situation, disposition, fatigue, and access to information, services, and healthcare professionals.
**Feelings**	Subjective emotional experiences that influence a person’s decision to initiate, maintain, or abandon health behaviors can favor or hinder the consolidation of health-promoting practices.
**Influences**	Influences are men’s cognitions and perceptions that influence behaviors. They can facilitate or hinder health-promoting behaviors. Men in social circles, women in family, work/school environments, fatherhood, support groups, healthcare professionals, and digital and virtual environments, such as social media, artificial intelligence, websites, and other virtual realities, positively or negatively influence health-promoting behaviors.
**Commitment to the action plan**	Interventions proposed by nursing and agreed upon with individuals to implement health-promoting behaviors should specify the time, place, and method of implementation, in addition to considering positive influences identified in individuals’ context. The included goals should, whenever possible, be shared with people who positively influence individuals’ adoption of health-promoting behaviors.
**Immediate demands**	These are behaviors that emerge into consciousness shortly before the adoption of a planned behavior, negatively interfering in the execution of the previously defined action plan.
**Predicted reactions**	Behaviors that men should establish as coping strategies to minimize the impact of immediate demands, ensuring greater adherence to the health behavior agreed upon with nursing professionals.

The Lighthouse Theory maintains the HPM dimensions because it constitutes a consolidated model in the literature on HP, including concepts fundamental to men’s health. The concept of disease is not part of the nursing metaparadigm; however, Pender’s approach to the HPM adds the concept of illness. Due to the close relationship between illness and Brazilian HP practices and the influence of this phenomenon on men’s HP, the derivation of this concept in the Lighthouse Theory was maintained. Monitoring MH-promoting behavior is the monitoring of the progress or lack thereof of the action plan. It should be developed jointly by nurses and men, in order to adjust pre-programmed actions to meet immediate demands. HP behavior is the end point or result of implementing the Lighthouse Theory.

**Figure 1:**
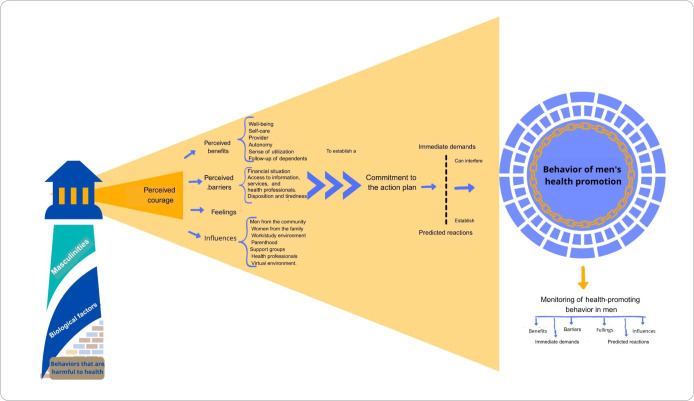
Modeling nursing metaparadigm concepts, central and associated concepts of middle-range theory for men’s health promotion, Cuiabá, Mato Grosso, Brazil, 2024

Perceived courage is the antithesis of fear, which cannot be used as a motivator for changes in health-promoting behaviors, but rather as a motivator for positive cognitions. Courage in men is commonly associated with harmful behaviors such as violence and recklessness, often fostered in this negative direction from early childhood. Thus, it is believed that courage must be mobilized toward the positive direction, mobilizing skills to confront health-damaging behaviors that often involve emotionally and morally difficult situations. It is this process of mobilizing courage that will determine for men the most important benefits, barriers, feelings, and influences for their current need for behavior change. The graphic representation of the Lighthouse Theory used principles of a mind map, with arrows and explanations (Figure 1).

The image representing health-promoting behavior is composed of a chain of links that demonstrates the man’s ongoing need for self-commitment to health-promoting behaviors. The surrounding sphere is composed of several fractures and exits, illustrating that behaviors can be remade and renewed, reaching other men. Just as men in frequent contact influence behavior change, a new health-promoting behavior can also motivate other men to change.

The path taken by the male population to obtain health-promoting behaviors must be monitored by nurses, who are supporters of the change processes and, therefore, must highlight the positive points of the process and use positive influences to achieve goals.

### Theory assumptions

The Lighthouse Theory presents ten assumptions, which should be interpreted as conceptions, truths relative to the theory and which need testing to be confirmed or refuted:

- All men possess perceived courage and are capable of mobilizing it toward health-promoting behaviors.
- Men have the ability to self-assess their perceived courage, including judging their own competencies and influences of masculinities on their health-promoting behaviors.
- Men engage in behaviors that provide perceived benefits they value, such as physical and mental well-being, self-care skills, staying active at work, and being sufficiently present in the upbringing and growth of children;
- Perceived barriers, such as lack of financial support, difficulties in accessing services, unqualified healthcare professionals and health information, and fatigue, can restrict the implementation of an action plan for health behaviors;
- Women in the family, groups in which the man is inserted, healthcare professionals and interaction with other men who exhibit behaviors that they consider healthy and admired, whether personally or professionally, are important sources of personal influence and can increase or decrease commitment and engagement in health-promoting behaviors.
- Work and leisure environments, as well as fatherhood, can increase commitment to health-promoting behaviors. The representation of masculinity present in the environment in which these men live and their relationship with healthcare influence the adoption of health-promoting behaviors.
- Commitment to an action plan is less likely to result in desired behavior when immediate demands arise over which the man has little control, requiring the activation of anticipated responses. Thus, both commitment to action and anticipated responses are influenced by masculinities;
- The action plan must address the virtual and real/traditional means of communication that people access to search for health information, in order to adapt them to the desired health-promoting behaviors;
- Health information provided by nurses tends to be more effective when it involves interest in men’s health and illness situation, time to embrace important elements, and guidance based on the men’s needs and interests.
- Younger men are more likely to make behavioral changes for HP, especially those related to mental health.

## DISCUSSION

In the field of nursing theories, this is the first MRT produced in the Brazilian Midwest. It is unprecedented, original, and has social impact and technological innovation in both the field of MH and theory development, aligned with the SDGs assumptions and the strengthening of nursing as a science. The technological innovation of Lighthouse Theory, with its soft-hard characteristics, according to Merhy^(16)^, rests on the idea of a new way for professionals, especially nurses, to carry out their MH promotion practices.

Thus, it advances in supporting men’s health demands, organizing knowledge to support nursing and health practices, in addition to describing how MH promotion occurs. Understanding what leads men and boys to prioritize care pathways and equitable versions of masculinities is essential to reformulating the feminist agenda on masculinities^(17)^, which has been unfounded in gender discourse.

In this context, it becomes important to understand the impact of harmful health behaviors on the male population. The 2019 report by the Promundo global organization, entitled “Masculine Norms and Men’s Health: Making the Connection”, highlights seven key health behaviors for men: poor diet, tobacco use, alcohol use, occupational risks, unsafe sex, drug use, and limited health-seeking behavior. These behaviors contribute to more than half of all premature male deaths and approximately 70% of male diseases^(18)^. Several of these involve sociobehavioral, environmental and economic aspects, which go beyond the possibilities of interventions by healthcare professionals.

However, it is possible to observe, in the daily life of health spaces where HP practices occur, that such key behaviors are often the focus of nursing action and, therefore, must be supported by a theoretical framework that is relevant and feasible for professionals and that meets the specificities of gender in our culture.

To this end, so-called gender-transformative strategies present promising paths toward implementing more inclusive and equitable practices. These approaches support individuals who identify as men to challenge and resist stereotypical male gender role norms that negatively impact health and well-being. They are increasingly recognized as a fundamental health and well-being strategy for this population^(19)^.

Highlighting this trend, research that aimed to map intervention studies that conceptualize masculinities identified six theoretical approaches used to support gender transformative strategies. These approaches helped operationalize and analytically transform masculinities, expanding and projecting individual behaviors and attitudes into broader analytical frameworks that can speak to structural changes in gender norms and relations^(20)^.

In the national context, this perspective gains strength in actions that specifically focus on men with activities aimed at HP, such as health education groups. When they have an approach focused on men, they can redefine the self-perception of masculinity^(21)^. This is where Lighthouse Theory gains strength as a theoretical framework for nursing. By finding support in Nola Pender’s HPM based on evidence of its effectiveness compared to traditional nursing consultation routines, Lighthouse Theory can promote better self-management of MH conditions, given that the care recipient is not always self-sufficient in assessing their health-promoting or non-health-promoting behaviors and establishing their MH goals. Thus, when nurses offer options for patients to choose which health behavior to adopt, patients’ sense of control and engagement in self-care increase^(17)^.

Thus, men’s active involvement in an action plan aimed at HP reveals itself to be a transformative mechanism, and can be considered a strategy that promotes a more equitable version of male beings, since behaviors far from care are still considered part of more traditional masculinities.

However, even when placed at the center of care and having their preferences respected, this development may not be immediate. Initially, individuals may have difficulty setting goals, and it is in this context that nurses act as a “supporter” in the process of self-assessing their behaviors and health condition, and in developing the care plan. Providing options allows individuals to choose and increase personal control over the proposed behavior^(22)^.

In the context of the SUS and aligned with objective five of the SDG, Lighthouse Theory can be applied in the partner’s prenatal consultations^(23)^, offering more robustness to the approach to the male public and using influences such as paternity, women in the family, healthcare professionals and benefits such as the sense of use and the provision of dependents for the adoption of health-promoting behaviors.

A randomized controlled clinical trial conducted in Iran that aimed to determine the effect of an educational intervention based on Pender’s HPM on adherence in patients with coronary artery disease recommended that nursing practices for this population should use it^(24)^. A similar result was found in a meta-analysis that aimed to better understand the effect of the HPM intervention on the physical and mental health of patients with diabetes mellitus. The HPM showed remarkable results, demonstrating a positive intervention effect on health knowledge, self-management behavior, and psychological function in patients with diabetes mellitus compared to the control group^(25)^.

Therefore, the conceptual elements of Lighthouse Theory, derived from the HPM, gain practical applicability when considering the meanings men attribute to their healthcare. It can guide nursing professionals to utilize the perceived benefits for the male population, such as autonomy in daily activities, monitoring the growth of dependents, and maintaining the role of family provider, with chronic conditions (diseases) being a driver of these behaviors. Thus, Lighthouse Theory, focused on MH, challenges the use of the biomedical care model alone, as it incorporates the assessment of behavioral factors linked to masculinities, moving away from the logic of medication prescription and focusing solely on disease.

### Study limitations

This study is limited in its applicability to diverse cultural contexts. The results and theoretical propositions presented here do not comprehensively address the specificities of HP within other sociocultural realities and international contexts. Further studies and approaches with other population groups, including diverse gender identities, are necessary. Furthermore, it was not possible to specifically address some social determinants of health, such as racial and socioeconomic markers, which are known to significantly differentiate health-promoting behaviors. Aspects related to violence, both in the private and public spheres, were also not addressed comprehensively, as they are directly related to the construction of masculinities and can significantly impact care practices and adherence to health-promoting actions. These dimensions, therefore, remain gaps to be explored in future studies to enrich and strengthen the theoretical and practical field of HP.

### Contributions to nursing

Lighthouse Theory demonstrates the importance of the male population for gender equality, the fifth PAHO SDG, and emphasizes nursing professionals in leading MH promotion. Within the disciplinary field of nursing, it is clear that Lighthouse Theory can guide professional practice by providing clarity on how MH promotion can be incorporated into nursing praxis. Furthermore, it positions Brazilian nursing at the forefront of MH promotion, establishing its leadership in the international landscape regarding research and practices focused on the male population.

It is important to emphasize that Lighthouse Theory can inform three areas related to nursing and MH. The first involves redefining policies and interventions aimed at promoting MH. The second focuses on advancing nursing and improving healthcare provided to Brazilian men, serving as a true beacon illuminating the path of nurses toward addressing the unfavorable situation facing the Brazilian and global male population. The third can theoretically support the development of a new nursing diagnosis, such as MH promotion, according to the NANDA International taxonomy and/or a terminological subset for MH promotion based on the International Classification for Nursing Practice. This focuses this population group on professional care and defines the role and place of nursing in the field of men’s health at the national and international levels.

## FINAL CONSIDERATIONS

The objective of this study is confirmed by producing a nursing MRT for MH promotion, composed of propositions involving a central concept, associated and metaparadigmatic concepts, and assumptions. These concepts are represented in a model, considering aspects related to masculinities and motivations that lead them to engage in behaviors aimed at improving their health, aligned with the SDGs. Lighthouse Theory can be applied by nursing professionals and other healthcare professionals in various contexts that promote self-care, the search for healthcare services, and the prevention of illnesses, such as primary healthcare settings, nursing offices in specialty outpatient clinics, and actions in workplaces, respecting the ethical and legal limits of each profession.

Furthermore, future versions of the theory may incorporate new empirical data, addressing topics such as violent behaviors associated with the male gender, the main causes of male morbidity and mortality, life cycles, race/color, and socioeconomic status, as well as the use of persuasive strategies and digital technology in care. As masculinities transform throughout history, Lighthouse Theory must be continually revisited to adapt to new realities.

## Data Availability

The research data are available within the article.
