# Supplementary material for: Middle-range theory for men’s health promotion: contributions to sustainable development
Source: Rev Bras Enferm. 2026 Feb 23;79:e20250216. doi: 10.1590/0034-7167-2025-0216 (PMC12933935; doi:10.1590/0034-7167-2025-0216)
Supplement: Translation

# Translation: Teoria de médio alcance para promoção da saúde do homem: contribuições para o desenvolvimento sustentável

**DOI:** 10.1590/0034-7167-2025-0216pt

**Authors:** Isabele Torquato Mozer Rosa, Aurea Christina de Paula Corrêa, Nuno Damácio de Carvalho Félix, Jeane Cristina Anschau Xavier Oliveira

**Affiliations:** I Universidade Federal de Mato Grosso. Cuiabá, Mato Grosso, Brasil; II IUniversidade Federal do Rencôcavo Baiano. Santo Antônio de Jesus, Bahia, Brasil.

**Keywords:** Teoria de Enfermagem, Promoção da Saúde, Saúde do Homem, Masculinidade, Formação de Conceito

## Abstract

**Objetivos:**

desenvolver uma teoria de médio alcance de enfermagem para promoção da saúde do homem alinhada ao Objetivo de Desenvolvimento Sustentável de igualdade de gênero.

**Métodos:**

estudo teórico indutivo-dedutivo, por meio da análise do Modelo de Promoção da Saúde, análise conceitual, finalidade, proposições, pressupostos e modelagem. A base empírica foi extraída da pesquisa nacional sobre a Política Nacional de Atenção Integral à Saúde do Homem na perspectiva da igualdade de gênero.

**Resultados:**

denominada de Teoria do Farol, a teoria é composta por 17 conceitos, dez proposições, quatro pressupostos, representados em uma modelagem, com a finalidade de descrever a promoção da saúde do homem que culmina em um plano de ação para comportamentos saudáveis, com vistas à igualdade no cuidado à saúde.

**Considerações Finais:**

a Teoria do Farol oferece um referencial teórico inovador para a enfermagem e saúde, fortalecendo práticas de promoção da saúde masculinas com descrição de comportamentos específicos.

## INTRODUÇÃO

A temática da saúde do homem (SH) e das masculinidades foi apresentada como um compromisso da Organização Pan-Americana da Saúde (OPAS) no Plano Estratégico 2014–2019^(1)^, e continua sendo um tema de enfoque nas ações rumo à saúde universal e à igualdade de género. Nesse contexto, a igualdade de género deve também envolver homens e os seus cuidados com saúde, uma vez que são considerados parte da solução para tais desigualdades. Em publicação recente, essa temática foi inserida no componente “saúde ao longo de todo ciclo de vida” do referido plano, para os anos de 2020 e 2025, alinhada também com a Agenda 2030 para o Desenvolvimento Sustentável^(2)^. Assim, o desenvolvimento do conhecimento teórico se alinha ao quinto Objetivo de Desenvolvimento Sustentável (ODS) (“Igualdade de género”), com potenciais benefícios ao apresentar caminhos para promoção da SH.

A enfermagem é considerada um corpo profissional importante para o alcance dos ODS. A preocupação em melhorar a qualidade da formação em enfermagem, com foco nas necessidades dos sistemas de saúde, acesso e cobertura universal de saúde, reforça a importância desta disciplina no alcance dos ODS^(3)^. A união de dois campos de investimentos dessa organização de saúde, base para a saúde pública das Américas, possibilita inúmeros benefícios para promoção da SH.

No contexto nacional, a SH se destacou como política pública a partir da instituição da Política Nacional de Atenção Integral à Saúde do Homem (PNAISH) em 2009, e, desde então, continua sendo a única nas América^(4)^. A discussão sobre masculinidades e a relação com as questões clínicas e epidemiológicas oferecem uma proposta, mesmo que tímida, inovadora para promoção e recuperação da saúde dessa população. Quinze anos após sua criação, a inclusão dos homens nos serviços como foco na promoção da saúde e a oferta de ações voltadas a este público continuam sendo tarefas desafiadoras para os profissionais de saúde, as quais demandam a estruturação de conhecimentos disciplinares para descrever o escopo da prática, em especial da enfermagem. Habitualmente, as ações de enfermagem à saúde masculina ainda se limitam à prevenção e ao tratamento do câncer de próstata em campanhas específicas^(5)^.

No cenário internacional, os homens estão envolvidos predominantemente nas testagens para infecções sexualmente transmissíveis e no pré-natal. Enfermeiras ainda não reconhecem os homens como sujeitos de seus cuidados, por falta de conhecimento sobre a reprodução masculina^(6)^. Evidências apontam que enfermeiros consideram insuficiente o conhecimento do conceito e definição da saúde masculina pela enfermagem, proveniente de carências na formação. Tais situações afetam a confiança em prestar cuidados ao público masculino neste espaço de atenção à saúde, refletindo também na percepção que os homens têm sobre o papel dos enfermeiros nos serviços de saúde^(7)^.

Nota-se escassez na literatura nacional e internacional de trabalhos que abordem o desenvolvimento de conhecimento teórico para a prática de enfermagem voltada para o cuidado à SH, expondo lacunas no conhecimento acerca da relação entre promoção da saúde (PS), SH e enfermagem. A prática profissional coaduna com os achados na literatura, apontando para a necessidade de um referencial teórico que apoie as práticas de enfermagem aos homens no contexto da PS.

Nesse contexto, inserem-se as teorias de enfermagem como alicerces da área da enfermagem fundamental, e não apenas podem, como devem, tornar-se um recurso para demonstrar caminhos que direcionam a prática profissional de enfermagem no âmbito da PS. No que tange ao desenvolvimento de teorias de enfermagem para este grupo populacional, tem-se a lacuna no conhecimento do campo disciplinar da enfermagem, em especial no que se refere às teorias de médio alcance (TMAs), consideradas como um meio para enfrentar o desafio de desenvolver um corpo de conhecimento próprio da disciplina que oriente a prática de enfermagem nos diversos contextos^(8)^, como versa o objeto de estudo em tela, contemplando conceitos que versam sobre as especificidades e subjetividades relacionadas à promoção da SH, podendo contribuir com a orientação da prática, do ensino, da gestão em serviços de enfermagem e da saúde.

## EOBJETIVOS

Desenvolver uma TMA de enfermagem para promoção da SH alinhada ao ODS de igualdade de género.

## MÉTODOS

### Aspectos éticos

Dispensou-se a submissão ao Comitê de Ética em Pesquisa, por se tratar de estudo de desenvolvimento teórico, mas foram respeitados os direitos autorais das publicações citadas, seguindo a Lei nº 9610/1998 que regulamenta tais práticas.

### Referencial teórico

O Modelo de Promoção da Saúde (MPS) de Nola Pender propõe a identificação de fatores que influenciam os comportamentos saudáveis para explorar os processos biopsicossociais que motivam indivíduos a se engajarem em comportamentos de saúde. O MPS baseia-se na teoria cognitiva social, defendendo que a PS promove estilos de vida e comportamentos que permitem que as pessoas maximizem seu potencial por meio de mudanças individuais, organizacionais e comunitárias, sendo o enfermeiro o catalisador desses processos. Ele é composto pela interrelação de três dimensões: as características e experiências individuais; os sentimentos e conhecimentos acerca do comportamento específico; e o comportamento de saúde desejável^(9)^.

### Tipo de estudo

Trata-se de estudo de desenvolvimento teórico, do tipo descritivo e natureza básica^(10)^, por meio do raciocínio de dedução-indução, a partir de dados empíricos da Pesquisa Nacional de Saúde do Homem para indução e fundamentada no MPS para dedução, gerando, por meio do processo de síntese, uma nova teoria. Esta teoria é resultante de tese de doutorado da Universidade Federal do Mato Grosso, tendo como cenário homens brasileiros e seus comportamentos de saúde^(11)^.

### Procedimentos metodológicos

A operacionalização deste estudo foi feita em quatro fases, conforme descrito por Meleis^(12)^: 1) escolha de um modelo teórico para explicar o fenômeno de interesse; 2) redefinir os conceitos da teoria; 3) síntese teórica; e 4) apresentação da nova teoria. Todo este processo é mental, e exige do teórico criatividade, abstração e reflexão. O pensamento focado em solução de problemas, a partir da interpretação e síntese dos achados, proporciona saltos intuitivos que auxiliarão no desenvolvimento da teoria e seus componentes.

O modelo teórico escolhido para explicar o fenômeno de interesse foi o MPS, por compreender que existem dimensões análogas entre o fenômeno de PS e a promoção da SH. Para tanto, utilizou-se a estrutura de análise de modelos de enfermagem de Jacqueline Fawcett^(13)^. Esse tipo de análise é um exame detalhado e sem julgamentos da teoria, que inclui determinar as origens da teoria, seu foco exclusivo e o conteúdo em si, como conceitos, afirmações, modelo e suposições. Para tanto, foi utilizada a obra literária *“Health Promotion in Nursing Practice”*, de autoria de Nola Pender, Carolyn Murdaugh e Mary Ann Parsons.

A partir da apreensão de uma estrutura teórica para sustentação, deu-se início à segunda fase, redefinição dos conceitos da teoria, realizada por meio de: a) análise do conceito segundo Walker e Avant^(14)^ para o conceito central da teoria de “promoção da SH”; b) derivação conceitual dos demais conceitos do MPS segundo Walker e Avant^(14)^. Para tanto, utilizaram-se os dados empíricos da mais recente pesquisa nacional realizada com homens brasileiros^(15)^. A teorização desses dados possibilitou dar voz à multifacetada população de homens brasileiros, fazendo um movimento inverso ao da construção da PNAISH, na qual os formuladores não faziam parte da base de interesse principal – o público masculino. Os elementos e conceitos da teoria são oriundos das interpretações das falas dos próprios homens, demonstrando o entendimento e suas necessidades para a PS, e valorizando principalmente as suas preocupações sobre cuidados de saúde.

A terceira fase, síntese teórica, foi realizada por meio da estratégia de síntese teórica^(14)^ devido à base de evidências empíricas, cujo objetivo foi integrar os conceitos redefinidos anteriormente em um arcabouço teórico coerente. Assim, os conceitos determinados na etapa anterior serviram como âncoras para o estabelecimento de suas relações a partir de afirmação de associações e/ou causalidade. Esse processo envolveu a formulação de proposições relacionais e a explicitação de pressupostos(14).

As proposições relacionais foram elaboradas para descrever as interações entre os conceitos centrais da Teoria do Farol. Para tanto, foi realizada: a) identificação de relações potenciais com base nos dados empíricos analisados, identificando padrões e associações entre os conceitos previamente definidos; b) formulação de proposições que foram redigidas a partir das relações identificadas utilizando uma linguagem clara e precisa. Cada proposição foi estruturada para refletir a natureza da relação (causal, correlacional, associativa) entre os conceitos envolvidos; c) validação teórica por meio da verificação de tais relações com a literatura existente para examinar sua consistência e plausibilidade teórica.

À medida que as proposições eram formuladas, *insights* de pressupostos eram registrados, sendo estruturados a partir das orientações de Walker e Avant(14). Para tanto, foram identificadas as crenças implícitas que orientaram a interpretação dos dados e a formulação das proposições para, a partir de então, serem redigidas como afirmações que delineiam as premissas aceitas como verdadeiras no contexto da teoria. Garantiu-se, assim, que os pressupostos fossem coerentes com os conceitos e proposições da teoria, evitando contradições ou inconsistências. Ao fim, foram estabelecidos dez pressupostos, afirmações aceitas como verdadeiras, que estabelecem as condições básicas sob as quais a teoria acontece.

O pictograma da teoria foi elaborado tomando por referência os elementos determinantes para PS masculina, e foi desenvolvido com o auxílio de uma plataforma *online* de *design* de comunicação visual, tendo como intuito ilustrar a prática de enfermagem no contexto da promoção da SH.

## RESULTADOS

### Contextualização da teoria

A TMA para promoção da SH, denominada de Teoria do Farol, recebeu este nome por duas razões. A primeira tem relação aos fatores biológicos, que tornam o ser homem único, associados às masculinidades. As expressões do ser homem no ambiente coletivo são a composição da luz que deve iluminar os comportamentos promotores de saúde e que necessariamente refletem nos demais. Masculinidades e fatores biológicos são conceitos considerados importantes na literatura sobre a SH. Todavia, vislumbrar a sua associação junto aos outros conceitos, da forma como propõe a Teoria do Farol, visa clarear para a disciplina da enfermagem uma nova forma de conduzir as práticas de PS de homens, principalmente no âmbito individual, tornando-se, assim, a segunda razão.

### Finalidades da teoria

A Teoria do Farol descreve a promoção da SH, a relação entre os comportamentos nocivos à saúde e às masculinidades, a coragem percebida, e a sua influência nos benefícios e barreiras percebidas, que culminam em um plano de ação envolvendo a participação de homens e profissionais de enfermagem na promoção de comportamentos de saudáveis, com vistas à igualdade no que se refere ao cuidado à saúde.

### Proposições e modelagem da teoria

A promoção da SH se refere a um processo contínuo e que pode ser mediado por profissionais de saúde a partir do reconhecimento dos elementos motivadores para o homem, estando relacionado às expressões de masculinidade do ambiente. Este é o conceito central, o qual possui uma definição derivada do MPS alinhada aos conceitos do metaparadigma da enfermagem e clarificada pela análise conceitual, apresentados como conceitos associados, conforme descrito no All men possess perceived courage and are Quadro 1.

**Quadro 1:** Proposições da teoria de médio alcance para promoção da saúde do homem, Cuiabá, Mato Grosso, Brasil, 2024

Conceitos	Proposições
**Conceito central**	
**Promoção da saúde do homem**	Comportamentos de busca de informações sobre saúde por meio de serviços de saúde e profissionais de saúde, outros homens de referência e meios de comunicação virtual, visando melhorar o seu bem-estar, prevenir agravos e doenças, e ter/manter ações autocuidado. Tais ações envolvem alimentação adequada, atividade física regular, hidratação, ações de higiene e repouso, previsões ambientais, práticas ligadas à cultura e religiosidade, cuidados estéticos e com a aparência física, baixa ingestão de álcool, ausência de tabaco, práticas sexuais seguras, calendário vacinal atualizado, olhar atento aos aspectos da saúde mental, e momentos de lazer.
**Conceitos metaparadigmáticos**	
**Pessoa**	Ser humano que se identifica como homem. Ele é moldado pelas suas experiências ao longo da vida junto ao contexto familiar, escolar, de trabalho e estudo, religiosidade, cultura e interações por meio virtual. As características individuais, as experiências de vida, o comportamento dos pares e o reforço positivo recebido pelo companheiro afetivo influenciam a modelagem dos comportamentos de saúde.
**Ambiente**	Contexto social, cultural, físico e virtual no qual o curso da vida dos homens se desenvolve, manipulado/trabalhado pelo indivíduo para criar um contexto positivo de sugestões e medidas que facilitarão comportamentos de melhoria da sua saúde, em especial quando recorrem a espaços que envolvem outros homens que possuem admiração.
**Enfermagem**	Profissão que atua como suporte/apoio na transformação de comportamentos da saúde do homem, junto a homens, mulheres, famílias e comunidade na qual o homem está inserido, desde que ele esteja disposto. Esta atuação visa criar um ambiente com condições favoráveis para expressão de saúde e bem-estar masculino, não sendo recomendado um papel de julgador por parte do profissional.
**Saúde**	No nível individual, é uma sensação de bem-estar adquirido por meio de comportamentos promotores de saúde, envolvendo a percepção de utilidade do homem no meio ao qual está inserido. As informações em saúde, sejam elas obtidas por meio virtual ou por profissionais de saúde, podem aumentar ou diminuir esta sensação de bem-estar.
**Conceitos associados**	
**Doença**	Acontecimentos, agudos ou crônicos, ao longo do ciclo de vida, podem ser um engajador de comportamentos promotores de saúde pelos homens.
**Fatores biológicos**	Idade, Índice de Massa Corporal, agilidade, estado hormonal, antecedentes de saúde pessoais e familiares.
**Masculinidades**	Comportamentos que identificam o ser homem na sociedade e conferem importância significativa ao comportamento existente e ao novo comportamento são influenciados por fatores como o nível educacional, socioeconômico, raça/etnia e psicológicos.
**Comportamentos nocivos à saúde**	Atitudes que devem ser mudadas com vistas à saúde e ao bem-estar, compostas pelos fatores biológicos, que não podem ser mudados e pelas masculinidades. Devem ser interpretados como alicerces dos comportamentos masculinos relativos à promoção da saúde e que devem/precisam ser mudados. São expressos na representação gráfica como um farol, pois sua luz ressoa nos demais conceitos, sendo a coragem percebida como o primeiro e mais caro à população masculina.
**Coragem percebida**	Julgamento das próprias habilidades relativas ao enfrentamento de situações emocionais ou moralmente difíceis para a mudança de um comportamento nocivo à saúde. Tal julgamento é diretamente influenciado pelas masculinidades e, portanto, estabelecer quais os benefícios, barreiras, sentimentos e influências é mais importante para o homem.
**Benefícios percebidos**	Representações mentais das consequências positivas ou reforçadoras de um comportamento, caracterizadas por bem-estar (ter e manter uma vida saudável e se sentir bem), autocuidado (possuir condições físicas e mentais de cuidar de si), ser provedor (percepção de prover financeiramente o sustento próprio e/ou familiar), autonomia (competência de gerir a própria vida e tomar decisões), senso de utilidade (percepção de ser capaz de resolver algo para si ou para alguém) e acompanhamento de descendente (participar ativamente do crescimento e desenvolvimento de filhos e netos).
**Barreiras percebidas**	Obstáculos percebidos por homens na adoção de comportamentos de promoção da saúde, caracterizadas pela situação financeira, disposição, cansaço e acesso a informações, serviços e profissionais de saúde.
**Sentimentos**	Experiências emocionais subjetivas que influenciam a decisão do homem em iniciar, manter ou abandonar comportamentos de saúde podem favorecer ou dificultar a consolidação de práticas promotoras de saúde.
**Influências**	Influências são cognições e percepções dos homens que envolvem os comportamentos. Eles podem facilitar ou impedir o comportamento promotor de saúde. Homens do convívio, mulheres da família, ambiente de trabalho/estudo, paternidade, grupos de apoio, profissionais de saúde e ambientes digitais e virtuais, como redes sociais, inteligência artificial, websites e outras realidades virtuais, influenciam positivamente ou negativamente os comportamentos promotores de saúde.
**Compromisso com o plano de ação**	Intervenções propostas pela enfermagem e pactuadas com o homem para efetivação do comportamento de promoção da saúde devem especificar tempo, local e modo de execução, além de contemplar influências positivas identificadas no contexto do indivíduo. As metas incluídas devem ser, sempre que possível, compartilhadas com pessoas que o influenciam de forma positiva na adoção de comportamentos promotores de saúde.
**Demandas imediatas**	São comportamentos que emergem a consciência pouco antes da adoção de um comportamento planejado, interferindo negativamente na execução do plano de ação previamente definido.
**Reações previstas**	Comportamentos que os homens devem estabelecer como estratégias de enfrentamento para minimizar o impacto das demandas imediatas, garantindo maior adesão ao comportamento de saúde acordado com o profissional de enfermagem.

A Teoria do Farol mantém as dimensões do MPS por se configurar um modelo consolidado na literatura sobre PS com a inclusão de conceitos fundamentais à saúde masculina. O conceito de doença não faz parte do metaparadigma da enfermagem; todavia, Pender no MPS acrescenta o conceito de doença. Por haver uma estreita relação da doença com as práticas de PS brasileiras e a influência desse acontecimento na PS de homens, manteve-se a derivação desse conceito na Teoria do Farol. Monitoramento do comportamento de promoção da SH é o acompanhamento do progresso ou não do plano de ação. Deve ser elaborado conjuntamente entre o enfermeiro e o homem, a fim de ajustar as ações pré-programadas diante das demandas imediatas. O comportamento de PS é o ponto final ou o resultado da implementação da Teoria do Farol.

A coragem percebida é a antítese do medo, que não pode ser usado como motivador para mudanças em comportamentos promotores de saúde, mas sim cognições positivas. A coragem no público masculino comumente está associada a comportamentos nocivos como os ligados à violência e à imprudência, frequentemente estimulada para esse pólo negativo desde a primeira infância. Assim, acredita-se que a coragem deve ser mobilizada para o polo positivo, mobilizando habilidades para o enfrentamento de comportamentos nocivos à saúde que geralmente envolvem situações emocionais e moralmente difíceis. É esse processo de mobilização da coragem que vai determinar para o homem os benefícios, as barreiras, os sentimentos e as influências mais importantes para sua necessidade atual de mudança de comportamento. A representação gráfica da Teoria do Farol usou princípios de um mapa mental, com setas e explicações (Figura 1).

**Figura 1:** Modelagem dos conceitos do metaparadigma da enfermagem, conceitos centrais e associados da teoria de médio alcance para promoção da saúde do homem, Cuiabá, Mato Grosso, Brasil, 2024

A imagem que representa o comportamento de promoção da SH é composta por uma corrente de elos que demonstra a necessidade contínua de comprometimento do homem consigo em relação aos comportamentos promotores de saúde. A esfera ao redor é composta de diversas fraturas e saídas, ilustrando que os comportamentos podem ser refeitos e renovados, atingindo outros homens. Assim como os homens do convívio frequente influenciam a mudança de comportamento, um novo comportamento de PS também pode motivar outros homens a mudanças.

O caminho percorrido pela população masculina para obtenção de comportamentos promotores de saúde deve ser acompanhado pelo enfermeiro, sendo esse um apoiador dos processos de mudança que, portanto, deve enaltecer os pontos positivos do processo e utilizar as influências positivas para o alcance de metas.

### Pressupostos da teoria

A Teoria do Farol apresenta dez pressupostos, que devem ser interpretados como concepções, verdades relativas à teoria e que precisam de testes para serem confirmadas ou refutadas:

- Todos os homens possuem coragem percebida e são capazes de mobilizá-las para comportamentos promotores de saúde.
- Os homens têm a capacidade de autoavaliar sua coragem percebida, incluindo o julgamento de suas próprias competências e influências das masculinidades em seus comportamentos promotores de saúde.
- Os homens se engajam em comportamentos pelos quais conseguem vislumbrar benefícios percebidos que valorizam, como o bem-estar físico e mental, a competência de se autocuidar, manter-se ativo no trabalho e estar suficientemente presente na criação e crescimento dos filhos;
- As barreiras percebidas, como falta de previsão financeira, dificuldades de acesso a serviços, profissionais de saúde e informações de saúde desqualificadas, e cansaço, podem restringir a execução do plano de ação para comportamentos de saúde;
- As mulheres da família, grupos nos quais o homem esteja inserido, profissionais de saúde e a convivência com outros homens que apresentam comportamentos que eles julgam saudáveis e admirados, seja pessoalmente ou profissionalmente, são importantes fontes de influência pessoal e que podem aumentar ou diminuir o comprometimento e engajamento em comportamentos promotores de saúde.
- Os ambientes de trabalho e de lazer, e o exercício da paternidade podem aumentar o comprometimento com comportamentos promotores de saúde. A representação de masculinidade presente no meio em que este homem está inserido e a sua relação com o cuidado de saúde influenciam a adoção de comportamentos promotores de saúde;
- O comprometimento com um plano de ação tem menor probabilidade de resultar no comportamento desejado quando ocorrem demandas imediatas sobre as quais o homem tem pouco controle, exigindo o acionamento das reações previstas. Assim, tanto o comprometimento com a ação quanto as reações previstas são influenciados pelas masculinidades;
- O plano de ação deve abordar os meios de comunicação virtuais e reais/tradicionais que o homem acessa para busca de informações de saúde, a fim de adequá-los para os comportamentos promotores de saúde desejados;
- As informações de saúde disponibilizadas por enfermeiros tendem a ser mais eficazes quando envolvem interesse na situação de saúde e doença do homem, tempo para acolher os elementos importantes e orientações a partir das necessidades e interesse dos homens.
- Homens mais jovens são mais propensos a realizar mudanças comportamentais para PS, em especial os relacionados à saúde mental.

## DISCUSSÃO

No campo das teorias de enfermagem, esta é a primeira TMA produzida no centro-oeste brasileiro, configurando-se como inédita, original, com impacto social e inovação tecnológica tanto no campo da SH quanto no de desenvolvimento de teorias, alinhadas aos pressupostos dos ODS e do fortalecimento da área da enfermagem enquanto ciência. A inovação tecnológica da Teoria do Farol, de característica leve-dura, segundo considerações de Merhy^(16)^, repousa na ideia de um novo modo de os profissionais, em especial os de enfermagem, realizarem suas práticas de promoção da SH.

Assim, avança no auxílio às demandas de saúde masculinas, organizando o conhecimento para fundamentar as práticas de enfermagem e saúde, além de descrever como ocorre a promoção da SH. Entender o que leva homens e meninos a priorizar caminhos de cuidado e versões equitativas de masculinidades é essencial para reformular a agenda feminista sobre masculinidades^(17)^ que foi fundamentada no discurso de gênero.

Nesse contexto, torna-se relevante compreender o impacto dos comportamentos nocivos à saúde na população masculina. O relatório de 2019 da organização Promundo Global, intitulado *“Masculine Norms and Men’s Health: Making the Connection”*, demonstra sete comportamentos-chave de saúde para os homens, como má alimentação, uso de tabaco, uso de álcool, riscos ocupacionais, sexo inseguro, uso de drogas e comportamento limitado de busca de saúde. Tais comportamentos contribuem para mais da metade de todas as mortes masculinas prematuras e cerca de 70% das doenças masculinas^(18)^. Vários desses envolvem aspectos sociocomportamentais, ambientais e econômicos, que vão além das possibilidades de intervenções dos profissionais de saúde.

Todavia, é possível observar, no cotidiano dos espaços de saúde onde ocorrem práticas de PS, que tais comportamentos-chave, frequentemente, são focos de atuação da enfermagem, e para tanto, devem ser sustentados por um arcabouço teórico pertinente e factível aos profissionais e que atenda às especificidades do gênero em nossa cultura.

Para tanto, as chamadas estratégias transformadoras de gênero se apresentam como caminhos promissores para a efetivação de práticas mais inclusivas e equitativas. Elas são abordagens que apoiam indivíduos identificados como homens a criticar e resistir às normas estereotipadas do papel de gênero masculino que afetam negativamente a saúde e o bem-estar, sendo cada vez mais reconhecidas como uma estratégia fundamental de PS para esta população^(19)^.

Evidenciando essa tendência, pesquisa que visou mapear os estudos de intervenção que trabalham conceitualmente as masculinidades identificou seis abordagens teóricas utilizadas para embasar estratégias transformadoras de gênero. Essas abordagens ajudaram a operacionalizar e transformar analiticamente as masculinidades, ampliando e projetando comportamentos e atitudes individuais em quadros de análise mais amplos que podem falar de mudanças estruturais nas normas e relações de gênero^(20)^.

No contexto nacional, essa perspectiva ganha força em ações que focam especificamente nos homens com atividades que visem à PS como grupos de educação em saúde, quando possuem uma abordagem focada em homens podem ressignificar a autopercepção de masculinidade^(21)^. É nesse ponto que a Teoria do Farol se fortalece enquanto suporte teórico para a enfermagem. Ao encontrar suporte no MPS de Nola Pender a partir das evidências de sua eficácia diante da rotina tradicional de consultas de enfermagem, a Teoria do Farol pode promover uma melhor capacidade de autogerenciamento das condições de SH, visto que nem sempre o sujeito de cuidado é autossuficiente para avaliar seus comportamentos que promovem ou não saúde e estabelecer suas metas para PS. Assim, quando o enfermeiro oferece opções para que o homem escolha qual comportamento de saúde é possível ser adotado, aumentam a sensação de controle sobre ela e o engajamento no seu autocuidado^(17)^.

Desse modo, o envolvimento ativo do homem em um plano de ação que vise à PS revela ser um mecanismo transformador, podendo ser considerado uma estratégia que potencializa uma versão mais equitativa do ser masculino, uma vez que comportamentos distantes do cuidado ainda são considerados parte das masculinidades mais tradicionais.

Entretanto, mesmo sendo colocado no centro do cuidado e tendo suas preferências respeitadas, essa construção pode não ser imediata. Inicialmente, os indivíduos podem ter dificuldade em estabelecer metas, e é nesse contexto que o enfermeiro atua como “apoiador” no processo de autoavaliação de seus comportamentos, de sua condição de saúde e na elaboração do plano de cuidados. Fornecer opções permite ao indivíduo escolher e aumentar o controle pessoal sobre o comportamento proposto^(22)^.

No contexto do SUS, e alinhado ao objetivo cinco do ODS, a Teoria do Farol pode ser aplicada nas consultas de pré-natal do parceiro^(23)^, oferecendo mais robustez à abordagem ao público masculino e utilizando influências como paternidade, mulheres da família, profissionais de saúde e benefícios como o senso de utilização e a provisão de dependentes para a adoção de comportamentos promotores de saúde.

Ensaio clínico controlado randomizado realizado no Irã, que teve como intuito determinar o efeito da intervenção educativa baseada no MPS de Pender na adesão de pacientes com doença arterial coronariana, recomendou que as práticas de enfermagem a essa população deveriam utilizá-lo^(24)^. Resultado semelhante foi encontrado em meta-análise que objetivou compreender melhor o efeito da intervenção do MPS na saúde física e mental de pacientes com diabetes mellitus também. O MPS apresentou resultados notáveis, demonstrando ter um bom efeito de intervenção no conhecimento de saúde, comportamento de autogestão e função psicológica de pacientes com diabetes mellitus em relação ao grupo controle^(25)^.

Portanto, os elementos conceituais da Teoria do Farol, derivados do MPS, ganham aplicabilidade prática ao considerar os significados atribuídos por homens ao cuidado à sua saúde. Ela pode orientar profissionais de enfermagem a utilizar os benefícios percebidos para a população masculina, como a autonomia nas atividades diárias, o acompanhamento do crescimento de dependentes e a manutenção de ser provedor de sua família, sendo a condição crônica (doença) um engajador desses comportamentos. Assim, a Teoria do Farol, voltada para a SH, contesta o uso apenas do modelo assistencial biomédico, pois agrega a avaliação de fatores comportamentais ligados às masculinidades, fugindo à lógica da prescrição medicamentosa e centrada somente na doença.

### Limitações do estudo

Este estudo possui como limitação a restrição quanto à sua aplicabilidade a contextos culturais diversos. Os resultados e proposições teóricas aqui apresentados não contemplam, de forma abrangente, as especificidades da promoção da SH inseridos em outras realidades socioculturais e contextos internacionais, sendo necessários estudos e aproximações com outros grupos populacionais, incluindo recortes de identidade de género variados. Ademais, não foi possível abordar com especificidade alguns determinantes sociais da saúde, como os marcadores raciais e socioeconômicos que, reconhecidamente, fazem diferenciações significativas nos comportamentos promotores de SH. Os aspectos relativos à violência, tanto no mundo privado quanto no público, também não foram abordados com a devida amplitude, uma vez que se relaciona diretamente com a construção das masculinidades, podendo impactar de maneira significativa as práticas de cuidado e a adesão a ações de PS. Tais dimensões, portanto, permanecem como lacunas a serem aprofundadas em estudos futuros, de modo a enriquecer e fortalecer o campo teórico-prático da promoção da SH.

### Contribuições para a área da enfermagem

A Teoria do Farol demonstra a importância da população masculina para a igualdade de género, quinto ODS da OPAS, além de sobrelevar o profissional de enfermagem na condução dos processos de promoção da SH. No campo disciplinar da enfermagem, vislumbra-se que a Teoria do Farol pode direcionar a prática profissional ao trazer clareza de como a promoção da SH pode ser incorporada na práxis de enfermagem. Para além, situa a enfermagem brasileira em uma posição de vanguarda no que se refere à promoção da SH, circunscrevendo sua liderança no panorama internacional referente às pesquisas e práticas voltadas à população masculina.

É pertinente salientar que a Teoria do Farol pode subsidiar três esferas relativas à enfermagem e à SH. O primeiro situa-se na redefinição de políticas e intervenções direcionadas à promoção da SH. O segundo repousa no avanço da enfermagem, bem como para a melhoria da assistência à saúde ofertada aos homens brasileiros, sendo um verdadeiro farol que ilumina o caminho dos enfermeiros com vistas ao enfrentamento do panorama desfavorável que vive a população masculina brasileira e mundial. O terceiro pode subsidiar, de forma teórica, a construção de um novo diagnóstico de enfermagem, o de promoção da SH, conforme a taxonomia NANDA Internacional, e/ou um subconjunto terminológico para promoção da SH nos moldes da Classificação Internacional para a Prática de Enfermagem, colocando esse grupo populacional no foco do cuidado profissional, demarcando, assim, o papel e o lugar da enfermagem no campo da saúde masculina no âmbito nacional e internacional.

## CONSIDERAÇÕES FINAIS

Confirma-se o objetivo deste estudo mediante a produção de uma TMA de enfermagem para promoção da SH composta por proposições envolvendo conceito central, conceitos associados e metaparadigmáticos, além de pressupostos, com a representação em uma modelagem, os quais consideram os aspectos relacionados às masculinidades e as motivações que os fazem se engajar em comportamentos direcionados à melhoria de sua saúde, alinhada ao ODS. A Teoria do Farol pode ser aplicada por profissionais de enfermagem e outros profissionais de saúde em diversos contextos que promovam o autocuidado, a busca por serviços de saúde e a prevenção de agravos, como espaços de Atenção Primária à Saúde, consultórios de enfermagem em ambulatórios de especialidades e ações em ambientes laborais, respeitando os limites ético-legais de cada profissão.

Além disso, futuras versões da teoria podem incorporar novos dados empíricos, abordando temas como comportamentos violentos associados ao género masculino, principais causas de morbimortalidade masculina, ciclos de vida, raça/cor e condição socioeconômica, além do uso de estratégias persuasivas e tecnologia digital no cuidado. Como as masculinidades se transformam ao longo da história, a Teoria do Farol deve ser continuamente revisitada para se adaptar às novas realidades.

## Data Availability

Os dados de pesquisa estão disponíveis no corpo do artigo.
